# A large scale bacterial attraction assay: A new quantitative bacterial migration assay suitable for genetic screens

**DOI:** 10.1371/journal.pone.0305037

**Published:** 2024-06-05

**Authors:** Thomas Quiroz Monnens, Alice Boulanger

**Affiliations:** LIPME, INRAE, CNRS, Université de Toulouse, Université Paul Sabatier, Castanet-Tolosan, France; Centre National de la Recherche Scientifique, Aix-Marseille Université, FRANCE

## Abstract

Bacteria use various motility mechanisms to explore their environments. Chemotaxis is the ability of a motile bacterial cell to direct its movement in response to chemical gradients. A number of methods have been developed and widely used to study chemotactic responses to chemoeffectors including capillary, agar plug, microscopic slide, and microfluidic assays. While valuable, these assays are primarily designed to monitor rapid chemotactic responses to chemoeffectors on a small scale, which poses challenges in collecting large quantities of attracted bacteria. Consequently, these setups are not ideal for experiments like forward genetic screens. To overcome this limitation, we developed the Large Scale Bacterial Attraction assay (LSBA), which relies on the use of a Nalgene™ Reusable Filter Unit and other materials commonly found in laboratories. We validate the LSBA by investigating chemoeffector kinetics in the setup and by using chemoattractants to quantify the chemotactic response of wild-type, and motility impaired strains of the plant pathogenic bacterium *Xanthomonas campestris* pv. *campestris* and the environmental bacterium *Shewanella oneidensis*. We show that the LSBA establishes a long lasting chemoeffector gradient, that the setup can be used to quantify bacterial migration over time and that the LSBA offers the possibility to collect high numbers of attracted bacteria, making it suitable for genetic screens.

## Introduction

Chemotactic-driven motility allows bacterial cells to adapt their movement to environmental conditions by sensing chemoeffector gradients. Chemotactic signaling is initiated by the recognition of chemoeffectors by dedicated transmembrane chemoreceptors called Methyl-accepting Chemotaxis Proteins (MCPs), which transduce the signal by forming tertiary complexes with different Che proteins. Chemoeffector recognition by the MCPs leads to a cascade of conformational changes of various proteins by methylation and phosphorylation, controlling the direction of cell movement [1]. The increasing number of sequenced bacterial genomes shows that although the central mechanisms of bacterial chemotaxis are highly conserved, there are added layers of complexity in a variety of species. For example, in *Pseudomonas aeruginosa* or *Myxococcus xanthus*, chemotactic signaling pathways are also involved in cellular functions other than taxis [2]. Furthermore, a recent study has shown that there are hundreds of families of MCPs across the bacterial kingdom with diverse ligand binding domains, suggesting that bacterial sensory mechanisms are highly diverse and selective [3]. Such findings open up new research opportunities in a variety of scientific and industrial fields, making the genetic determinants of chemotaxis, MCP specificity, and the response to different chemoeffectors topics which are relevant to modern research.

**Fig 1:**
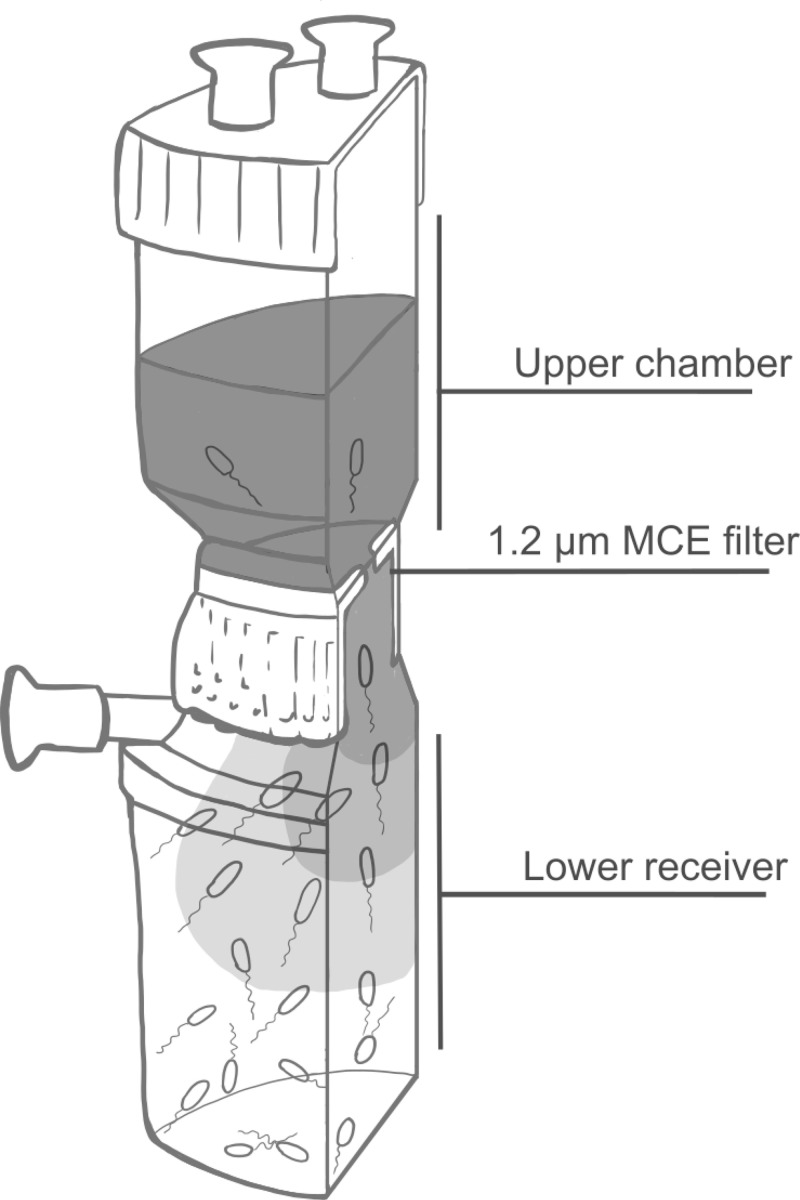
Schematic representation of the chemoeffector gradient and bacterial movement in the large-scale attraction assay. This drawing represents a Nalgene™ Reusable Filter Unit filled with bacterial suspension in the lower receiver and chemoattractant (in dark grey) in the upper chamber. Chemoattractant will slowly diffuse from the upper chamber to the lower receiver. Chemoattractants stimulate bacterial movement from the lower receiver through the 1.2 μm mixed cellulose ester filter to the upper chamber.

Various methodologies, requiring skilled manual handling, have been developed and are widely used to investigate the mechanisms underlying bacterial chemotaxis in response to chemoeffectors. These methodologies include the capillary [4], agar plug [5], microfluidic systems [6, 7] and microscopic slide assays [8]. Chemotactic response occurs within seconds, resulting in a series of run-and-tumble events. Consequently, the above methods are designed to measure chemotactic responses on a small scale and on a short timescale. Therefore, they do not allow for the collection of large populations of bacteria attracted by specific chemoeffectors. However, to conduct genetic screens to identify genes involved in bacterial attraction, it is necessary to collect a large number of attracted cells. This underscores the need for the development of methodologies capable of efficiently collecting and analyzing large bacterial populations responding to specific chemoeffectors. To address this requirement, we have designed the Large Scale Bacterial Attraction Assay (LSBA, Fig 1). Briefly, a Nalgene™ Reusable Filter Unit is filled with a non-chemotactic buffer separated by a 1.2 μm hydrophilic mixed cellulose ester filter. Bacterial cells are added to the lower receiver of the Filter Unit through the outlet port, after which a chemoeffector can be added to the buffer in the upper chamber. Chemoeffectors will diffuse from the upper chamber through the filter towards the bottom compartment, creating a concentration gradient. Chemoattractants will attract bacteria, prompting them to migrate against gravity, through the filter to the upper chamber.

To test the performance of the LSBA, we first quantified the diffusion of glucose and methylene blue from the upper chamber to the lower receiver to gain a better understanding of chemoeffector diffusion in our system. We then quantified bacterial attraction of the wild type and a motility mutant of *Xanthomonas campestris* pv. *campestris* (*Xcc*) strain 8004 towards glucose, glutamate and cauliflower xylem sap over a two days period of time. *Xcc* is the causal agent of black rot disease of cultivated (e.g. cabbages, cauliflower) and wild Brassicaceae (e.g. Arabidopsis) [9]. We also measured attraction towards L-malate of the environmental bacterium *Shewanella oneidensis* (*So*) wild type strain MR1 (Bordi *et al*., 2003). As a control, we also monitored the potential growth of both species in the solutions used to attract cells. Collectively, our results indicate that the LSBA establishes a long-lasting chemoeffector gradient and that it is a suitable system for the quantification of bacterial attraction. Moreover, the LSBA allows for the collection of high number of attracted bacterial cells, making it suitable for forward genetic screens.

## Materials and methods

The protocol described in this peer-reviewed article is published on protocols.io (https://doi.org/10.17504/protocols.io.n2bvjn65bgk5/v1) and is included for printing purposes as S1 File. Diffusion of glucose and methylene blue in LSBA system is available in S2 File.

## Results

### The LSBA allows the establishment of robust and long-lasting chemoeffectors gradients

Establishing the optimal incubation time of the experiment is of particular importance to collect high numbers of attracted bacteria. A brief incubation time may yield insufficient migrating bacteria while a too long incubation time might lead to the equilibrium of chemoeffector concentration gradients, potentially elevating false positives resulting from random migration. Predicting the time required for the LSBA to attain chemoeffector equilibrium poses a challenge due to the multifaceted nature of factors impacting the diffusion speed. These factors encompass the diffusion coefficient of the chemoeffector, the dimensions of both the chemoeffector and filter pores, as well as potential interactions between the chemoeffector and the filter material. To gain a better understanding of chemoeffector dynamics in the LSBA, we measured the diffusion of glucose and methylene blue from the upper chamber to the lower receiver at 28°C. We choose these two compounds as they differ in size (180.16 and 319.85 g/mol respectively) and their concentrations in a solution can be easily measured (S2 File). As the lower receiver is sealed, it is not possible to measure the concentration of the compounds directly. We therefore tracked the concentration of either glucose or methylene blue in the upper chamber and deduced the concentration in the lower receiver using this formula: ${\text{C}}_{\left(\text{lower}\;\text{receiver}\right)}^{t}{=[\text{n}}_{\left(\text{upper}\;\text{chamber}\right)}^{i}{–\text{n}}_{\left(\text{upper}\;\text{chamber}\right)}^{t}{]/\text{V}}_{\left(\text{lower}\;\text{receiver}\right)}$

$${\text{n}}_{\left(\text{upper}\;\text{chamber}\right)}=\left(\text{OD}-\text{Yintercept}\right)/\left(\text{slope}\right)$$

**Fig 2:**
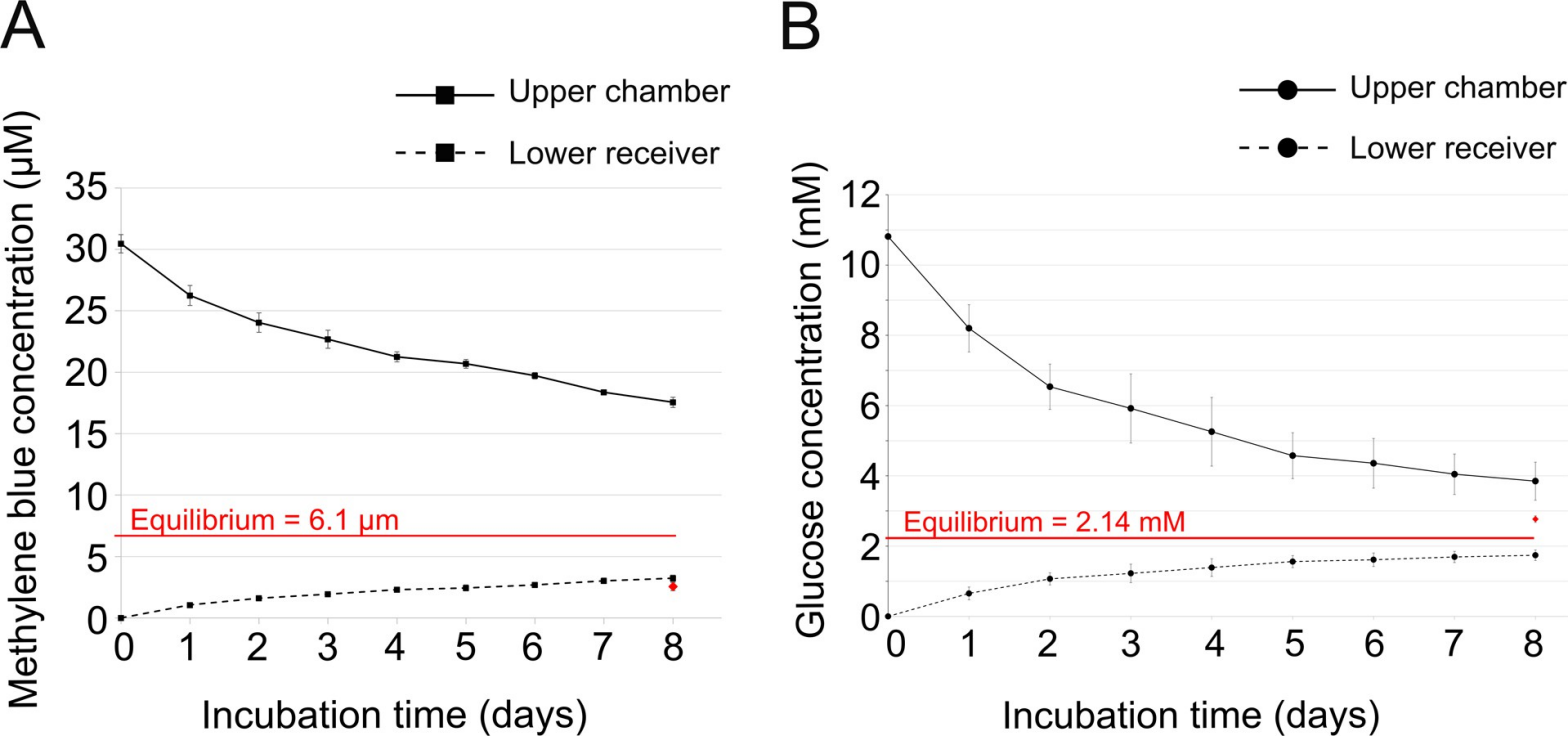
Diffusion of methylene blue and glucose in the LSBA. (**A**) The change in glucose concentration in each compartment of LSBA over a period of eight days at 28°C. The concentration of glucose in the upper chamber was determined by using Benedict’s quantitative reagent followed by absorbance at 740 nm (S2 File). The distribution of glucose was then determined by calculating the absolute amount of glucose diffusing from the upper chamber to the lower receiver and calculating the resulting difference in concentration between the upper chamber and the lower receiver (S2 File). (**B**) The change in the proportion of methylene blue in the upper chamber compared to the lower receiver over a period of eight days at 28°C. The concentration of methylene blue in the upper chamber at the start of the experiment was 30 μM. The concentration of methylene blue in the upper chamber was determined by absorbance at 652 nm over a period of eight days (S2 File). The distribution of methylene blue was then determined in the same way as for glucose. For A and B, the red diamond observed for 8 days of incubation correspond to the concentration of methylene blue and glucose measured directly from lower receiver respectively. Values are calculated as the mean of three biological replicates.

Methylene blue, initially present at 30 μM in the upper chamber, diffused into the bottom receiver, resulting in a concentration of 17.55 μM after the same incubation period (Fig 2A and S2 File). Notably, this concentration remained considerably higher than the equilibrium concentration of 6.1 μM. Furthermore, the concentration was also measured directly from lower receiver at the end of the experiment. The value obtained (2.55 μM) is close to the one deduced (Fig 2B, red diamond). Similarly, glucose, with an initial concentration of 10 mM in the upper chamber, exhibited a gradual decrease to 3.85 mM after eight days of incubation (Fig 2B and S2 File). Consequently, the deduced concentration in the bottom receiver increased to 1.74 mM, indicative of diffusion from the upper chamber. However, the measured glucose concentration in the lower receiver after eight days of incubation is higher than the one deduced from measured in upper chamber with a value of around 2.77 mM. This value is higher than the equilibrium concentration of approximately 2 mM (Fig 2B, red diamond). Despite this result, the curves obtained indicate that the diffusion dynamics of glucose and methylene blue in this system establish prolonged chemoeffector gradients. This indicates that, for chemoeffectors with diffusion speeds similar to glucose and methylene blue, the LSBA establishes long-lasting chemoeffector gradients.

### The chemotactic response of *Xcc* and *So* to chemoeffectors in the LSBA

**Fig 3:**
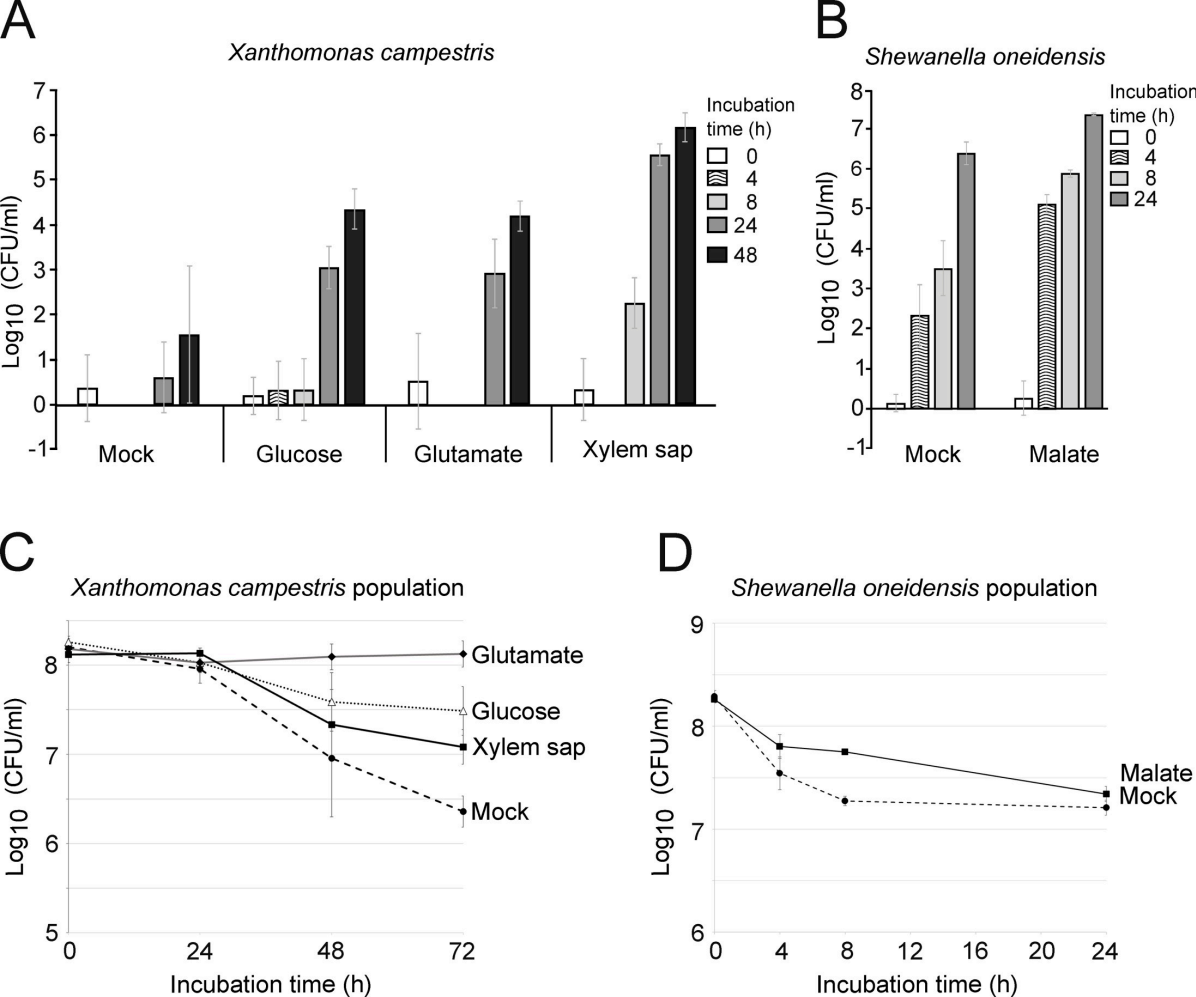
Chemotactic response in the LSBA. (**A**) The chemotactic response of *Xcc* strain 8004::GUS*-GFP* towards 1 mM MgCl_2_ as a control (Mock) or towards Cauliflower’s xylem sap (1% v/v), glucose (10 mM) and glutamate (10 mM) diluted in a 1 mM MgCl_2_ buffer was followed using LSBA over a two-days period of time. Histograms represent the mean of four biological replicates. (**B**) The chemotactic response of *So* MR-1 strain towards LM buffer (Mock) or Malate (10 mM). For A and B, bacteria were suspended in Mock buffer at a final concentration of 10^8^ cells/ml in the lower receiver. The number of bacteria migrating over time was determined by plating 100 μl of bacterial suspension collected from the upper chamber on either MOKA rich medium supplemented with rifampicin (50 μg per ml) and pimaricin (30 μg per ml) or LB supplemented with rifampicin (50 μg per ml) respectively. T0 is taken 30 minutes after adding the bacteria to the system. Histograms represent the mean of four biological replicates. **(C)** Population size of *Xcc* starting at an OD_600nm_ of 0.1 in 100 ml 1 mM MgCl_2_ (Mock) and 1 mM MgCl_2_ with the tested chemoeffectors after incubation at 28°C. The population size was determined by plating aliquots on MOKA solid medium and subsequent viability counting. Values are calculated as the mean of three biological replicates. (**D**) Population size of *So* in LM medium (Mock) and LM medium with 10 mM malate after incubation at 28°C. The *So* population size was determined by plating aliquots on LB solid medium and subsequent viability counting. Values are calculated as the mean of three biological replicates.

The chemotactic response of *Xcc* strain 8004::GUS*-GFP* [carrying the point mutations inactivating the catalytic sites of the β-glucuronidase and GFP proteins, made in a *Xcc* 8004::GUS-GFP strain background [10]] towards different chemoeffectors was quantified using the LSBA (Fig 3A). This assay was carried out over a two-day period using three different chemoeffectors; glucose, glutamate [one of the major amino acids of xylem sap consumed by *Xcc* [11]] both at a final concentration of 10 mM, or cauliflower xylem sap [1% v/v, *Brassica oleracea* var. *botrytis* cv. Clovis (L.)]. *Brassica oleracea*’s xylem sap contains relatively low concentrations of chemicals, with sugars and amino acids typically below 1 mM [11], and a 1% diluted xylem sap used in our assay assures low attractant concentrations. All attractants were diluted in 1 mM MgCl_2_ buffer as this is the common physiological buffer used to resuspend many *Xanthomonoas* species including *Xcc* [10]. Addition of the chosen chemoeffectors did not alter the pH of the 1 mM MgCl_2_ solution. A solution of 1 mM MgCl_2_ was used as a negative control (Mock). During these two days of incubation, we assumed that the chemoeffector equilibrium between the upper chamber and lower receiver for small molecules like glucose was not reached yet (Fig 2). For each experiment, a verification of the system was performed 30 minutes after adding bacteria in lower receiver by measuring potential contamination of the upper chamber. High bacterial counts at this time indicate a damaged filter or improper step-up. This corresponds to our time point zero in Fig 3.

Over the course of the experiment, a substantial increase in *Xcc* population was observed in the upper chamber supplied with 1% xylem sap, with an approximate six log increase in bacterial count (Fig 3A).

Notably, a significant rise in bacterial abundance was also detected in chambers supplemented with either 10 mM glutamate or glucose, contrasting with minimal changes observed in the mock condition. The efficiency of our system was also evaluated with a different bacterial species, specifically the wild type *Shewanella oneidensis* (*So*) MR-1 strain [12]. Migration of *So* MR-1 cells towards malate, a documented attractant for *So* MR-1 [13, 14], was quantified (Fig 3B). Prior to experimentation, *So* bacterial cells were resuspended in LM medium adjusted to pH 7.2 (Mock), in accordance with the established physiological conditions commonly employed in *So* chemotaxis studies [14]. Subsequently, malate was added to the solution to achieve a final concentration of 10 mM, necessitating the buffering of the solution to pH 7.2. Results demonstrated a rapid migration of *So* cells to the upper chamber, as evidenced by a significant increase in population size observed after only four hours of incubation in the presence of attractants. A difference of around two hundred folds was observed between the mock condition and the presence of malate after only four hours of incubation. However, in contrast to observations with *Xcc*, a gradual increase in population size over time was noted in the mock condition. This phenomenon may be attributed to *So*’s ability to perform aerotaxis towards oxygen gradients, a mechanism documented in previous studies [13]. To assess the potential influence of bacterial growth on the observed migration patterns of *Xcc* and *So* in the upper chamber, both bacterial strains population size was monitored at 28°C overtime with an initial OD_600nm_ of 0.1 in 100 ml of their respective buffers. The change in population size was determined by quantifying the colony-forming units (CFU) per ml through viability counting (Fig 3C and 3D). Remarkably, no discernible growth was detected for either *Xcc* or *So* during the relevant incubation periods. On the contrary, population size decreased over time refuting the hypothesis that bacterial growth is responsible for the population increase in the upper chamber. Altogether, these results underscore the effectiveness of the LSBA in measuring bacterial attraction toward chemoeffectors.

**Fig 4:**
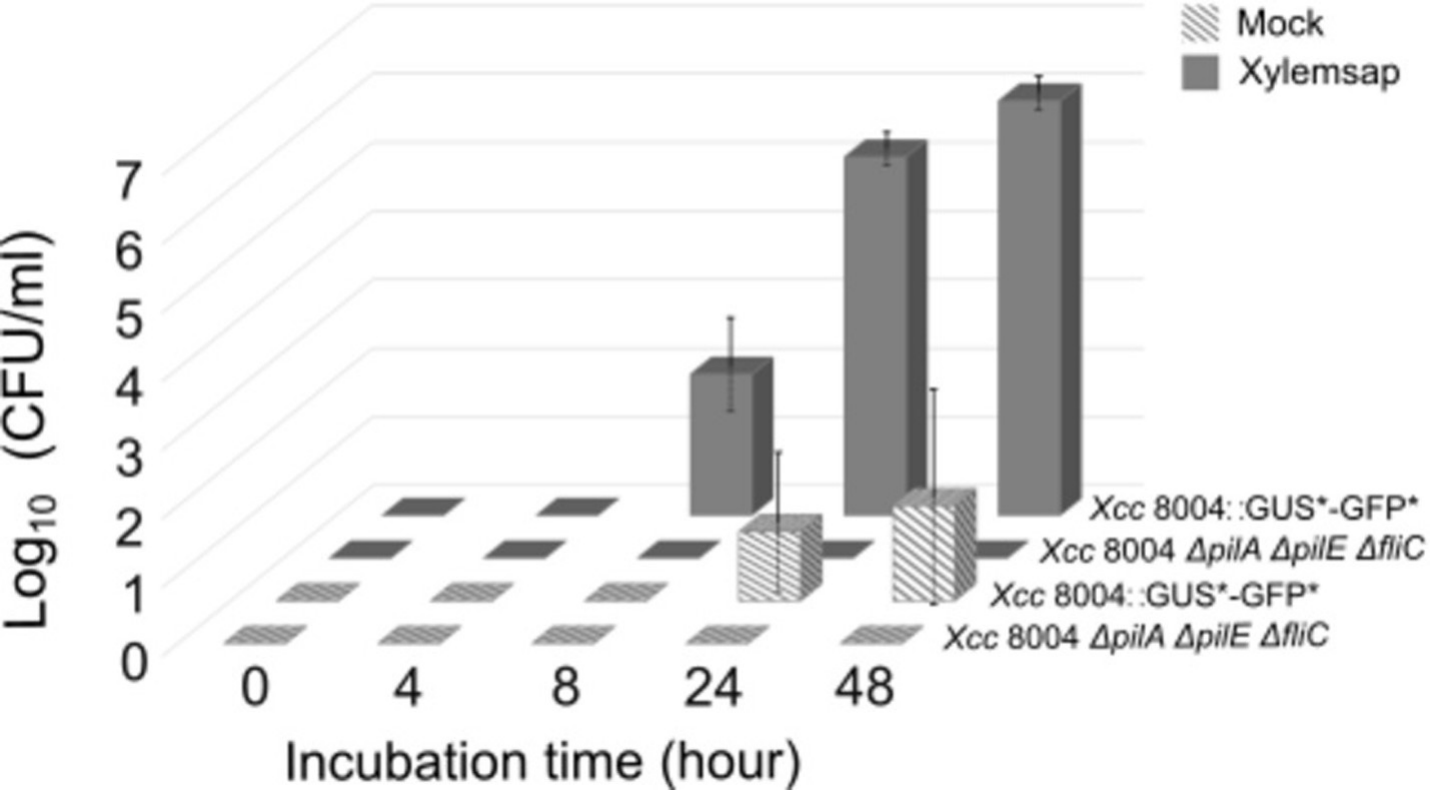
Competition assay between *Xcc* strain 8004::GUS*-GFP* and a multiple knockout motility mutant (*Xcc* strain 8004::GUS-GFPΔ*pilA*Δ*pilE*Δ*fliC*) in a 1:1 ratio. The total bacterial concentration at the start of the experiment in the lower receiver was 10^8^ cells/ml. The number of wild-type and motility-compromised *Xcc* bacteria migrating to the upper chamber toward mock or 1% v/v xylem sap was determined by plating 100 μl of the upper chamber on solid MOKA medium supplemented with 200 μM X-gluc. Only the motility mutant form blue colonies [10]. Histograms represent the mean of four biological replicates.

To use the LSBA for genetic screen, it is important to verify that for a heterogenous population comprising cells impaired for migration toward chemoeffectors, only wild-type cells will be able to be attracted to the upper chamber. In a competition assay, we compared cells migration towards 1% v/v diluted cauliflower xylem sap between *Xcc* strain 8004::GUS*-GFP* and a motility impaired *Xcc* strain 8004::GUS-GFP mixed in a 1:1 ratio (Fig 4). The motility mutant is deleted for both the *fliC* flagellin and the *pilA* and *pilE* pillins [10]. The results obtained show that the motility impaired strain is never able to reach the upper chamber even after 48 hours of incubation. The LSBA appears to discriminate between migrating and non-migrating cells.

## Discussion

In this study, we introduced the LSBA, a novel experimental setup that can be used to quantify bacterial attraction towards chemoeffectors and facilitate the collection of sufficient bacterial quantities for forward genetic screens. Our findings highlight several key aspects of the LSBA’s functionality and utility. Firstly, we demonstrated the LSBA’s capacity to establish a long-lasting chemoeffector gradient, and that the LSBA can quantitatively assay the effect of chemoeffectors on a population of bacteria over a period of time. Notably, our experimental conditions precluded significant bacterial growth in the upper chamber, emphasizing the distinct contributions of motility versus growth dynamics to observed migration patterns. Furthermore, our results underscored the essential role of motility in facilitating efficient *Xcc* migration through the filter to the upper chamber of the LSBA. Next step, would be to test if a well characterized strong chemotaxis mutant of *Xcc* is also impaired in its ability to migrate towards the attractants (CheY our CheW mutant for example). Unfortunately, such mutant is not yet available. Moreover, the *Xcc* 8004 genomes encodes multiple paralogues of different Che genes, and it is unknown which are required for chemotaxis, making it difficult to identify candidate gens of such validations [15].

Importantly, our study revealed a marked disparity in bacterial population sizes between conditions with and without the presence of attractants, underscoring the sensitivity and specificity of the LSBA in detecting chemoeffector-mediated responses.

In two other studies were *Xcc* chemotaxis towards 10 mM glucose was studied using capillary assays, it was found that the number of bacteria attracted by glucose was only 2 [16] or 3 [17] times higher than the number of bacteria in the capillaries without attractants. In the LSBA, the number of bacteria attracted by the same concentration of glucose is 20 times higher than the numbers of bacteria in the upper chambers without attractant (Fig 3).

It is imperative to acknowledge the need for adaptation of the LSBA to accommodate the unique characteristics of each bacterial species under investigation. As microbial behaviors and responses vary across species, customization of experimental parameters and conditions is essential to ensure the reproducibility and applicability of results across diverse microbial systems.

This specific setup was designed and optimized for use with a 1.2 μm mixed cellulose ester filter. *Xcc* and *So*, the model bacteria used to validate the LSBA, have an average diameter of 0.5–1.0 μm. This means that both are able to cross the filter. However, other species of bacteria might be bigger in size, which would prevent them from passing such filter. One could use a filter with a bigger pore size for other bacteria. However, it should be considered that this would also affect the diffusion kinetics of chemoeffectors in the LSBA.

For our research topic, we designed the LSBA to work specifically for chemoattractants. We did not investigate the chemotactic response in relation to chemorepellents. However, we believe that this setup could also be used to evaluate the effect of chemorepellents on a specific population of bacteria. For this purpose, chemorepellents can be mixed with bacteria in the lower receiver and repelled bacteria can be collected over time in the upper chamber. However, also here it should be considered that the diffusion kinetics might be different compared to adding chemoeffectors to the upper chamber.

The number of attracted bacteria per ml in the LSBA is in the same order of magnitude as the number of attracted bacteria per ml in capillary assays [17]. However, whereas a capillary test generally contains only a few microliters, the LSBA contains 100 ml, which allows for the collection of high numbers of attracted bacteria. Using xylem sap as an attractant for example yields 10^8^ *Xcc* cells using a single LSBA. This means that the LSBA can also be used for experiments for which high numbers of bacteria are needed, such as genetic screens aimed at identifying genetic determinants of bacterial migration towards specific compounds. In light of our findings, the LSBA emerges as a preferred tool for conducting screen assays in conjunction with well-characterized chemotaxis assays. The complementary nature of the LSBA and established chemotaxis assays offer a synergistic approach to elucidate the complexities of bacterial chemotaxis. Integrating the LSBA into screening protocols provides researchers with a powerful tool to identify genetic determinants involved in bacterial attraction and to unravel the contributions of individual genes to chemotactic behavior. As such, the LSBA represents a valuable addition to the arsenal of tools available for studying bacterial chemotaxis, offering researchers a versatile platform for uncovering novel insights into microbial behavior and environmental interactions.

## Supporting information

S1 File Protocol for the LSCA. (PDF)

S2 File Diffusion of methylene blue and glucose in the LSCA. (DOCX)
