# Supplementary material for: A large scale bacterial attraction assay: A new quantitative bacterial migration assay suitable for genetic screens
Source: PLoS One. 2024 Jun 5;19(6):e0305037. doi: 10.1371/journal.pone.0305037 (PMC11152280; doi:10.1371/journal.pone.0305037)
Supplement: S1 File — (PDF) [file pone.0305037.s001.pdf]

May 24, 2024

A large scale bacterial attraction assay: a new quantitative bacterial migration assay suitable for genetic screens

DOI
dx.doi.org/10.17504/protocols.io.n2bvjn65bgk5/v1

Thomas Quiroz Monnens[1], Alice Boulanger[1]

[1]LIPME, Université de Toulouse, INRAE, CNRS, Université Paul Sabatier, 31320, Castanet-Tolosan, France

Boulanger Alice
université Paul sabatier - toulouse III

OPEN ACCESS

DOI: dx.doi.org/10.17504/protocols.io.n2bvjn65bgk5/v1

Collection Citation: Thomas Quiroz Monnens, Alice Boulanger 2024. A large scale bacterial attraction assay: a new quantitative bacterial migration assay suitable for genetic screens. **protocols.io** https://dx.doi.org/10.17504/protocols.io.n2bvjn65bgk5/v1

License: This is an open access collection distributed under the terms of the **Creative Commons Attribution License**, which permits unrestricted use, distribution, and reproduction in any medium, provided the original author and source are credited

Protocol status: Working
We use this collection and it's working

Created: May 03, 2024

Last Modified: May 24, 2024

Collection Integer ID: 99639

Funders Acknowledgement:
ANR JCJC
Grant ID: ANR-19-CE20-JCJC-0014-01

Disclaimer

This protocol was peer reviewed and accepted for publication in Plos One protocol

Abstract

Bacteria use various motility mechanisms to explore their environments. Chemotaxis is the ability of a motile bacterial cell to direct its movement in response to chemical gradients. A number of methods have been developed and widely used to study chemotactic responses to chemoeffectors including capillary, agar plug, microscopic slide, and microfluidic assays. While valuable, these assays are primarily designed to monitor rapid chemotactic responses to chemoeffectors on a small scale, which poses challenges in collecting large quantities of attracted bacteria. Consequently, these setups are not ideal for experiments like forward genetic screens. To overcome this limitation, we developed the Large Scale Bacterial Attraction assay (LSBA), which relies on the use of a Nalgene™ Reusable Filter Unit and other materials commonly found in laboratories. We validate the LSBA by investigating chemoeffector kinetics in the setup and by using chemoattractants to quantify the chemotactic response of wild-type, and motility impaired strains of the plant pathogenic bacterium *Xanthomonas campestris* pv. *campestris* and the environmental bacterium *Shewanella oneidensis*. We show that the LSBA establishes a long lasting chemoeffector gradient, that the setup can be used to quantify bacterial migration over time and that the LSBA offers the possibility to collect high numbers of attracted bacteria, making it suitable for genetic screens.

Disclaimer

This protocol was peer reviewed and accepted for publication in Plos One protocol

Files

SEARCH

Protocol

NAME

Protocol preparation of bacterial cells

VERSION 1

CREATED BY

Boulanger Alice
université Paul sabatier - toulouse III

OPEN →

Protocol

NAME

Protocol LSBA setup

VERSION 1

CREATED BY

Boulanger Alice
université Paul sabatier - toulouse III

OPEN →

Protocol references

1. Blanvillain S, Meyer D, Boulanger A, Lautier M, Guynet C, Denancé N, et al. Plant carbohydrate scavenging through tonB-dependent receptors: a feature shared by phytopathogenic and aquatic bacteria. PloS One. 2007;2: e224. doi:10.1371/journal.pone.0000224

2. Boyeldieu A, Poli J-P, Ali Chaouche A, Fierobe H-P, Giudici-Orticoni M-T, Méjean V, et al. Multiple detection of both attractants and repellents by the dCache-chemoreceptor SO_1056 of Shewanella oneidensis. FEBS J. 2022;289: 6752–6766. doi:10.1111/febs.16548

3. Cerutti A, Jauneau A, Auriac M-C, Lauber E, Martinez Y, Chiarenza S, et al. Immunity at Cauliflower Hydathodes Controls Systemic Infection by Xanthomonas campestris pv campestris. Plant Physiol. 2017;174: 700–716. doi:10.1104/pp.16.01852

4. Luneau JS, Baudin M, Quiroz Monnens T, Carrère S, Bouchez O, Jardinaud M-F, et al. Genome-wide identification of fitness determinants in the Xanthomonas campestris bacterial pathogen during early stages of plant infection. New Phytol. 2022. doi:10.1111/nph.18313

5. Dugé de Bernonville T, Noël LD, SanCristobal M, Danoun S, Becker A, Soreau P, et al. Transcriptional reprogramming and phenotypical changes associated with growth of Xanthomonas campestris pv. campestris in cabbage xylem sap. FEMS Microbiol Ecol. 2014;89: 527–541. doi:10.1111/1574-6941.12345
