# Supplementary material for: A large scale bacterial attraction assay: A new quantitative bacterial migration assay suitable for genetic screens
Source: PLoS One. 2024 Jun 5;19(6):e0305037. doi: 10.1371/journal.pone.0305037 (PMC11152280; doi:10.1371/journal.pone.0305037)
Supplement: S2 File — (DOCX) [file pone.0305037.s002.docx]

Protocol to measure the diffusion of methylene blue and glucose in the LSBA

The chemoeffector dynamic disequilibrium between the upper chamber and lower receiver influence the ideal sampling window of the LSBA. Predicting the time required to reach the equilibrium of the chemoeffector is challenging because of the various factors that influence the speed of diffusion in this system. To gain a better understanding of the chemoeffector dynamics in the LSBA, we measured the diffusion of glucose and methylene blue from the upper chamber to the lower receiver at 28°C. Here we describe how to measure the diffusion of these compounds in the LSBA in case modifications of the system are made and a new estimation of diffusion is required. The diffusion of methylene blue is measured using an initial concentration of 31.26 μM in non-chemotactic buffer. We choose this concentration as for 100 μl of this solution it is possible to measure the methylene blue concentration by absorbance in a colorimeter. The diffusion of glucose is measured using an initial concentration of 10 mM in non-chemotactic buffer. We choose this concentration because it corresponds to the concentration used for the chemoeffector response assay of *Xanthomonas campestris* pv. *campestris* (*Xcc*). The concentration of glucose is measured using a homemade Benedict’s solution and a subsequent absorbance measurement in a colorimeter [1].

Protocol to measure concentration of methylene blue in the LSBA

Estimated preparation time is around 20 minutes for the LSBA (as described in S1 File) and an additional 5 minutes per sampling and measurement of time point. The assay takes eight days.

1. Setup the LSBA as according to the protocol described in S1 File.
2. Add methylene blue to the top compartment of the LSBA to reach a final concentration of 31.26 μM in 100 ml and mix by pipetting. Be careful not to touch the filter with the pipette tip, as it is delicate.
3. Each day, sample three times 100 μl of upper chamber solution and measure its absorbance in a Greiner 96 wells flat bottom cell culture plate at a wavelength of 652 nm.
4. Calculate the absolute amount of remaining methylene blue in the upper chamber of the LSBA using the standard curve (example given in Figure 1A). A new standard curve is prepared each day of sampling. The amount in the bottom compartment is that which moved from the top compartment.
5. Calculate the deduced concentration of methylene blue in the bottom compartment of the LSBA by:

C^t^_(lower receiver)_ = [n^i^_(upper chamber)_ – n^t^_(upper chamber)_]/ V_(lower receiver)_

n_(upper chamber)_ = (OD -Yintercept)/(slope)

Materials

1. Methylene blue solution at initial concentration of 31.26 mM (1% w/v).
2. Greiner 96 wells flat bottom cell culture plate (or any other plate suitable for colorimetry)

Protocol to measure concentration of glucose in the LSBA

Estimated preparation time is around 20 minutes for the LSBA (as described in S1 File) and around 30 minutes to prepare 100 ml of Benedict’s solution. It takes an additional 15 minutes per sampling time point. The assay takes eight days.

1. Setup the LSBA as according to the protocol described in S1 File.
2. Add glucose to the upper chamber to reach a final concentration of 10 mM in 100 ml and mix by pipetting. Be careful not to touch the filter with the pipette tip, as it is delicate.
3. Each day, sample three times 200 μl of upper chamber solution and mix it in a 3:1 (600 μl and 200 μl) ratio with a homemade Benedict’s quantitative solution (see materials) in an Eppendorf tube.
4. Incubate the mixture for ten minutes at 99°C
5. Centrifuge the solution for 2 minutes at 10.000 g.
6. Take 100 ul of the supernatant and measure its absorbance in a Greiner 96 wells flat bottom cell culture plate at a wavelength of 740 nm.
7. Calculate the absolute amount of remaining glucose in the upper chamber of the LSBA using the standard curve (example given in Figure 1B). A new standard curve was prepared for each repetition.
8. Calculate the deduced concentration of methylene blue in the bottom compartment of the LSBA by:

C^t^_(lower receiver)_ = [n^i^_(upper chamber)_ – n^t^_(upper chamber)_]/ V_(lower receiver)_

n_(upper chamber)_ = (OD -Yintercept)/(slope)

Materials

1. 1 M glucose solution.
2. Greiner 96 wells flat bottom cell culture plate (or any other plate suitable for colorimetry)
3. Homemade Benedict’s quantitative solution:
   1. Make solution 1 (10 g C_6_H_7_NaO_7_, 3.25 g anhydrous Na_2_CO_3_, 0.25g KSCN in 50 ml of dH_2_0).
   2. Make solution 2 (0.9 g Copper sulphate.5H_2_O in 50 ml of dH_2_0)
   3. Slowly add solution 2 to solution 1 under stirring.
   4. Add 0.013 g of K_3_[Fe(CN)_6_] to the mixed solutions.
4. Eppendorf tubes


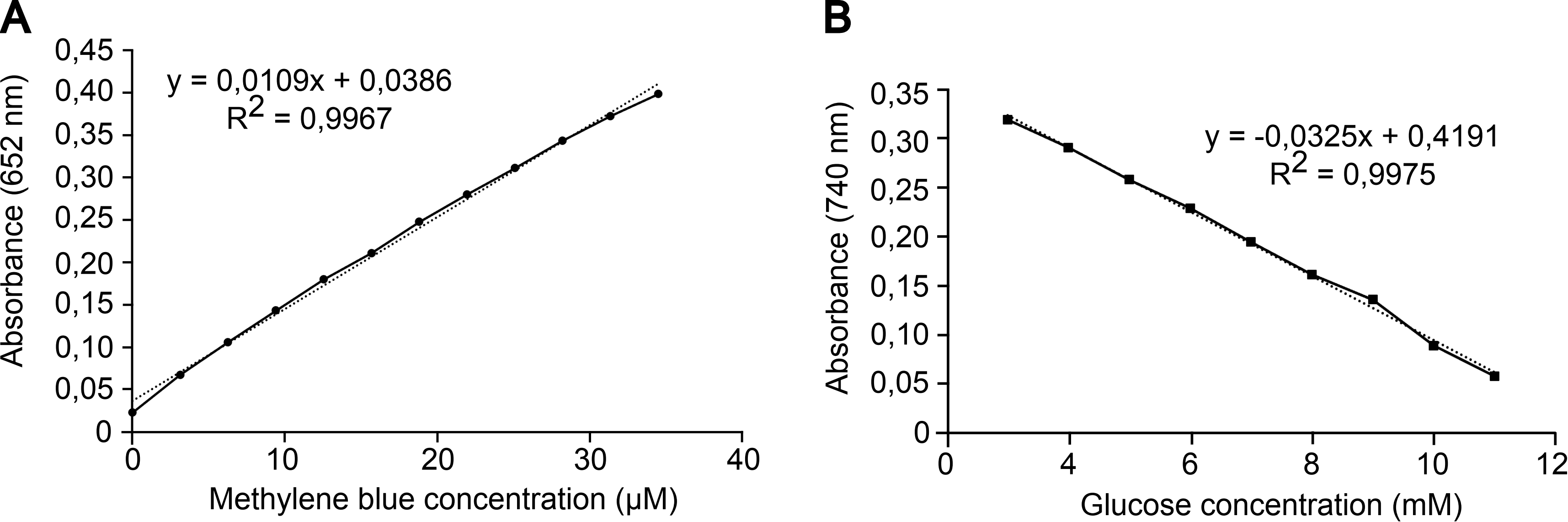


**Fig 1. Examples of standard curves for methylene blue and glucose in the concentrations relevant to this study.** (A) Standard curve of methylene blue absorbance. Absorption of 0 to 31.26 μM methylene blue in non-chemotactic buffer (1 mM MgCl_2_) was measured for 100 μl in a Greiner 96 wells flat bottom cell culture plate at a wavelength of 652 nm. (B) Standard curve of Benedict’s quantitative solution after incubation with glucose solutions ranging from 0 to 11 mM at 740 nm.

References

1. Hernández-López A, Sánchez Félix DA, Zuñiga Sierra Z, García Bravo I, Dinkova TD, Avila-Alejandre AX. Quantification of Reducing Sugars Based on the Qualitative Technique of Benedict. ACS Omega. 2020;5: 32403–32410. doi:10.1021/acsomega.0c04467
